# Distinct metabolic signatures define key HIV-1 clinical phenotypes and reveal targets beyond viral suppression

**DOI:** 10.3389/fimmu.2026.1979386

**Published:** 2026-10-23

**Authors:** Jacobo Alonso-Domínguez, Raquel Lama, Inés Martínez-Barros, Aida López-López, Alexandre Pérez-González, Ezequiel Ruiz-Mateos, Luis F López-Cortés, Iris Samarra, Salvador Fernandez-Arroyo, Anna Rull, Javier Martinez-Picado, Roberto Güerri-Fernández, Mayte Coiras, Pepa Galindo, Karenina Antelo Cuéllar, Víctor Asensi, Eva Poveda

**Affiliations:** 1 Virology and Pathogenesis, Galicia Sur Health Research Institute (IIS Galicia Sur), SERGAS-UVIGO, Vigo, Spain; 2 Department of Pharmacy, Complexo Hospitalario Universitario de Vigo (CHUVI), Sergas, Vigo, Spain; 3 Internal Medicine Department, Complexo Hospitalario Universitario de Vigo (CHUVI), Sergas, Vigo, Spain; 4 Clinical Unit of Infectious Diseases, Microbiology and Parasitology, Institute of Biomedicine of Seville (IBiS), Virgen del Rocio University Hospital, Spanish National Research Council (CSIC), University of Seville, Seville, Spain; 5 Eurecat, Centre Tecnològic de Catalunya, Centre for Omic Sciences (Joint Unit Eurecat - Universitat Rovira i Virgili), Unique Scientific and Technical Infrastructure (ICTS), Reus, Spain; 6 Institut de Recerca Biomèdica Catalunya Sud, Hospital Universitari de Tarragona Joan XXIII (HJ23), Universitat Rovira i Virgili (URV), Tarragona, Spain; 7 CIBERINFEC, Carlos III Health Institute, Madrid, Spain; 8 Germans Trias i Pujol Research Institute, Badalona, Barcelona, Spain; 9 IrsiCaixa, Badalona, Barcelona, Spain; 10 University of Vic-Central University of Catalonia (UVic-UCC), Vic, Spain; 11 Catalan Institution for Research and Advanced Studies (ICREA), Barcelona, Spain; 12 HIV-STD Division, Group for Research in Viral Infections (GRIV), Hospital del Mar Research Institute, Barcelona, Spain; 13 Department of Medicine and Life Sciences (MELIS), Univ. Pompeu Fabra, Barcelona, Spain; 14 Immunopathology and Viral Reservoir Unit, National Center of Microbiology, Instituto de Salud Carlos III, Majadahonda, Madrid, Spain; 15 Unidad de Enfermedades Infecciosas, Hospital Clínico Universitario de Valencia, Valencia, Spain; 16 Unidad de Enfermedades Infecciosas, Hospital Universitario de Dénia, Denia, Spain; 17 Infectious Diseases Unit, Hospital Universitario Central de Asturias and Group of Translational Research in Infectious Diseases, Instituto de Investigación Sanitaria del Principado de Asturias (ISPA), Oviedo, Spain

**Keywords:** antiretroviral therapy, elite controllers, HIV, immune non-responders, metabolomics

## Abstract

**Introduction:**

Despite effective antiretroviral therapy (ART), metabolic dysfunction persists in people with HIV (PWH) and may contribute to immune dysregulation and non-AIDS comorbidities. However, the metabolomic profiles associated with untreated infection, immune recovery, immune non-response, and spontaneous viral control remain incompletely defined.

**Methods:**

Plasma metabolomics was performed in 140 participants: HIV-negative controls (n=30), treatment-naive individuals (n=30), ART-treated immune responders (IR; n=30), immune non-responders (INR; n=20), and elite controllers (n=30), subdivided into persistent (PC; n=15) and transient controllers (TC; n=15). Targeted and semi-targeted LC-MS and GC-MS analyses were combined with differential metabolite, over-representation, and quantitative enrichment analyses.

**Results:**

The extent of metabolic disruption differed markedly across phenotypes. Treatment-naive individuals showed 142 altered metabolites, followed by INR (114), TC (68), and PC (28), whereas IR showed only 4. Untreated infection was characterized by sphingolipid depletion, triglyceride accumulation, and mitochondrial pathway alterations. Despite comparable ART exposure, INR retained pronounced eicosanoid, amino acid, and energy metabolism abnormalities, whereas IR exhibited near-normalization, indicating that residual dysfunction was associated with impaired immune reconstitution rather than ART itself. Elite controllers displayed distinct metabolic adaptations: PC showed increased acylcarnitines and enrichment of pyruvate metabolism, the urea cycle, and the Warburg effect, whereas TC exhibited broader carbon and nitrogen metabolic perturbations before loss of spontaneous viral control. Arachidonic acid metabolism remained altered across all HIV phenotypes.

**Conclusions:**

Distinct metabolomic signatures characterize the major viroimmunological phenotypes of HIV infection. Effective ART combined with immune reconstitution was associated with metabolic normalization, while spontaneous viral control revealed pathways potentially relevant to immunological stability.

## 1 Introduction

The advent of antiretroviral therapy (ART) has transformed HIV infection from a fatal diagnosis to a manageable chronic condition, significantly reducing morbidity and mortality (1). However, persistent immune dysfunction and metabolic disturbances remain critical challenges, even among individuals with sustained viral suppression. While ART effectively suppresses viral replication, up to 30% of treated people with HIV fail to restore CD4^+^ T-cell counts to protective levels (>350 cells/μl), a condition termed immune non-response (INR) (2). Conversely, a rare subset of individuals, known as elite controllers (ECs), naturally maintain undetectable viral suppression without ART, offering unique insights into mechanisms of viral control (3). Despite advances in understanding immunological disparities, the metabolic underpinnings of these heterogeneous clinical outcomes remain poorly characterized.

Metabolomics, a high-throughput approach to identify small-molecule metabolites, has emerged as a powerful tool to unravel pathophysiological pathways in chronic diseases, including HIV (4). Dysregulated lipid metabolism, mitochondrial dysfunction, and altered amino acid profiles have been linked to chronic inflammation, immune exhaustion, and comorbidities in people with HIV (PWH) (5). However, while prior metabolomic studies in PWH have typically compared discrete groups (e.g., treatment-naive vs. ART-treated, immune responders vs. non-responders, or transient vs. persistent controllers), our study uniquely encompasses the full spectrum of viroimmunological statuses in HIV infection within a single cohort. Elucidating metabolic signatures across these phenotypes may reveal novel biomarkers of immune recovery, viral control, disease progression, and help to make interventions for preventing comorbidities.

This study characterized the plasma metabolomic profiles of five carefully defined groups: (1) HIV-negative controls, (2) ART-naive participants, (3) ART-treated participants with viral suppression and optimum CD4^+^ recovery (>350 CD4^+^ T-cell counts after two years of ART), (4) INRs (viral suppression but CD4^+^ <350 cells/μl after ≥2 years of ART), and (5) ECs, who are able to achieve undetectable viral load levels in plasma in the absence of ART and who were further stratified into persistent controllers (PCs) and transient controllers (TCs). Each phenotype was compared with HIV-negative controls to define the systemic metabolic disruptions associated with viral replication, impaired immune reconstitution and spontaneous viral control, and complementary pairwise analyses were then used to isolate the metabolites and pathways specific to each clinical condition. TC samples, collected prior to loss of control, offer a unique window into early metabolic shifts preceding HIV-disease progression.

By defining cohort-specific metabolic signatures, this study aimed to unravel how HIV infection, antiretroviral therapy, and natural viral control remodel host metabolism and to identify a characteristic metabolic fingerprint for each phenotype. Through the integration of these comparisons, we sought to address key knowledge gaps that could ultimately serve as a basis for optimizing therapeutic strategies and precision monitoring protocols.

## 2 Material and methods

### 2.1 Study participants

The study comprised 140 participants divided into five groups: (1) HIV-negative controls (n = 30); (2) treatment-naive participants recently diagnosed with HIV (n = 30); (3) ART-treated participants (median treatment duration: 11.4 years) with sustained viral suppression and normalized CD4^+^ T-cell counts (>350 cells/μl, n = 30), referred to as immune responders (IR); (4) immune non-responders (INRs, n = 20), defined as participants on ART for ≥2 years with undetectable viral loads but persistent low CD4^+^ T-cell levels (<350 cells/μl); and (5) elite controllers (ECs, n = 30) who achieve undetectable viral load levels in plasma in the absence of ART, subdivided into persistent controllers (PCs, n = 15), who naturally maintained viral suppression permanently without ART, and transient controllers (TCs, n = 15) who eventually lose the control of the virus with detectable viral load levels, whose plasma samples were collected a median of 30 [14–48] months before viral control was lost. Control and IR groups were provided by the IISGS (Instituto de Investigación Sanitaria Galicia Sur) Biobank. The INR group was recruited from Hospital Universitario Virgen del Rocío (Sevilla, Spain). The naive and EC groups were recruited by CoRIS and ECRIS cohorts with their samples provided by the Spanish HIV BioBank. The CoRIS cohort has been described in detail elsewhere (6).

### 2.2 Data collection and processing protocol

Anonymized demographic and clinical data, including age, sex, CD4^+^ and CD8^+^ T-cell counts, viral load, ART regimens, and biochemical parameters, together with the corresponding plasma samples, were provided for all participants in accordance with ethical protocols approved by the relevant institutional review boards. Written informed consent was obtained from all participants prior to sample collection and inclusion in the study. All samples were processed and stored at −80 °C until analysis to minimize pre-analytical variability.

Whole blood was collected in EDTA tubes and processed to isolate plasma. Samples were centrifuged using a Ficoll density gradient to separate peripheral blood mononuclear cells (PBMCs), and the plasma fraction was carefully aspirated. Plasma was then aliquoted into cryovials and stored at -80 °C until metabolomic analysis.

### 2.3 Targeted and semi-targeted metabolomics

Plasma metabolomic profiling was conducted using complementary platforms based on liquid (LC) and gas (GC) chromatography coupled to triple quadrupole (QqQ) or quadrupole time-of-flight (QTOF) mass spectrometer (MS). A targeted metabolomics approach was followed to analyze polar metabolites, including TCA cycle intermediates. In this case, metabolites were derivatized with methoxyamine and N-methyl-N-trimethylsilyltrifluoroacetamide +1% trimethylchlorosilane after methanol extraction and analyzed by GC-QTOF-MS equipped with a DB-5MS column (Agilent Technologies, Santa Clara, CA, USA) under helium flow and a temperature ramp (60 °C to 320 °C). For acylcarnitine and lipidomics analyses, a semi-targeted metabolomics approach was followed and involving two different extraction strategies: 1) Folch-based extraction using chloroform:methanol (2:1, v/v) for a comprehensive lipidome coverage via LC-QTOF-MS working in positive mode and using a Kinetex EVO C18 column (Phenomenex, Torrance, CA, USA) and a gradient of ternary mobile phases consisting on water, methanol and 2-propanol with 10 mM ammonium formate and 0.1% formic acid. The identification of lipid species was performed by Agilent MassHunter Lipid Annotator 1.0 software. [M+H]+ and [M+NH4]+ precursors were considered. To enable product-ion spectral matching against in silico generated databases, the lipid annotation used data acquisition at the MS/MS level; 2) Acylcarnitines and lipidic intermediates were extracted by mixing plasma with methanol and analyzed via LC-QqQ-MS using the multiple reaction monitoring mode. For acylcarnitines, a Kinetex Polar C18 column (Phenomenex) with 0.1% formic acid-methanol/water gradient was used for chromatographic separation and MS working in positive mode. For lipidic intermediates, a CORTECS C18 column (Waters Corp., Milford, MA, USA) and a gradient of water + 0.05% acetic acid and acetonitrile were used for chromatographic separation and MS working in negative mode.

Across all analytical platforms, a total of 421metabolites were reliably detected and quantified and constituted the reference background for all over-representation analyses. In all cases, selected labeled internal standards were included for monitoring, controlling and correction of analytical signal drift and ensure data quality, reproducibility and accuracy. Raw chromatogram data (.d format) are publicly available in BioStudies (https://www.ebi.ac.uk/biostudies/), under accession number S-BSST3409.

### 2.4 Metabolite and pathway analysis

All metabolomic data processing and statistical analyses were performed using MetaboAnalyst 6.0 with the integrated Small Molecule Pathway Database (SMPDB) version 2.0. Prior to analysis, features with >50 % missing values across samples were excluded. Remaining missing values were imputed using the limit of detection (LoD) approach, where each missing entry was replaced with one-fifth of the minimum positive value detected for that variable across the entire dataset. To mitigate variability, normalization was performed using the median, and the data were log10 transformed to stabilize variance. Auto scaling (mean-centering followed by division by the standard deviation) was applied to ensure comparability across metabolites.

### 2.5 Individual metabolite analyses

For each clinical subgroup – naive (n=30), immune non-responders (INR, n=20), immune responders (IR, n=30), transient controllers (TC, n=15) and persistent controllers (PC, n=15) – pairwise comparisons against HIV-negative controls (n=30) were conducted using volcano plots. Statistical significance was defined by a false discovery rate (FDR) < 0.05 and an absolute fold-change threshold > 1.5. Additionally, sparse partial least squares discriminant analysis (sPLS-DA) was applied to each pairwise comparison to identify the most discriminant metabolites while reducing overfitting. To further interpret the underlying structure driving the separation, loadings for the first two components (loading 1 and loading 2) were extracted. These loadings provided the direction and magnitude of the contribution of each metabolite to each component.

### 2.6 Pathway enrichment analyses

Pathway-level interpretation was performed using two complementary approaches: First, Over-Representation Analysis (ORA) was performed on the set of metabolites that met the volcano-plot criteria (FDR < 0.05, |FC| > 1.5). The reference background was the complete set of metabolites detected by our analytical platforms. Enrichment was tested against the Small Molecule Pathway Database (SMPDB), and pathways with at least two matched metabolites were retained. Statistical significance was established after false discovery rate correction for multiple comparisons, with an FDR threshold of 0.05. Second, Quantitative Enrichment Analysis (QEA) was carried out on the full, non-filtered metabolite dataset for each pairwise comparison. The same SMPDB pathway sets were used, and significance was evaluated using a global test statistic with FDR adjustment. In both ORA and QEA, pathways with an FDR < 0.05 were considered significantly enriched.

The marked difference in the number of significant pathways identified by ORA and QEA reflects the distinct assumptions of the two approaches. ORA evaluates only the subset of metabolites exceeding the volcano-plot thresholds against the full set of detected metabolites, and therefore requires a substantial number of individually significant hits within a given pathway. QEA, in contrast, incorporates the direction and magnitude of change of all quantified metabolites, and consequently detects coordinated but individually subthreshold perturbations. The two methods should therefore be interpreted as complementary: ORA identifies pathways dominated by large individual effects, whereas QEA captures diffuse, pathway-wide remodeling. Throughout this study, pathways supported by both approaches were considered the most robust. Complete lists of differentially abundant metabolites and pathway enrichment analyses for all comparisons are provided in **Supplementary Table 1**–**6**.

### 2.7 Ethical considerations

The study protocol was approved by the Ethics Committee of Galicia (CEIm-G) (Ref: 2019/447). The studies were conducted in accordance with the local legislation and institutional requirements. The CoRIS cohort was approved by the Research Ethic Committee of the Gregorio Marañón Hospital. The participants provided their written informed consent to participate in this study. Biobank samples and associated data were handled in strict compliance with national regulations on personal data protection and biomedical research. All datasets used for analysis were pseudonymized to ensure participant confidentiality and data security.

## 3 Results

### 3.1 Characteristics of the study population

The study comprised 140 participants across four distinct viroimmunological groups and HIV-negative controls, as detailed in the Methods. The demographic and clinical characteristics of the six study cohorts are summarized in **Table 1**. The groups were well-balanced for sex distribution, with a predominance of males across all HIV positive phenotypes. As expected, INR and IR participants had comparable ART duration (median 11.6 and 11.4 years, respectively), yet exhibited profoundly different CD4^+^ T cell trajectories: INR displayed persistently low CD4^+^ counts (median 203 cells/μl), whereas IR achieved normalization (median 855 cells/μl). Elite controllers (TC and PC) showed undetectable viral loads without ART, with PC having higher CD4^+^ counts and CD4/CD8 ratios than TC. Notably, TC and PC also differed in nadir CD4^+^ and years since diagnosis, consistent with their divergent clinical outcomes. These clinical data confirm the accurate classification of the study phenotypes and provide the foundation for the metabolomic analyses presented below.

**Table 1:** Clinical and epidemiological characteristics of study cohorts.

	Control (n = 30)	ART-naive (n = 30)	Immune non-responders (n = 20)	Immune responders (n = 30)	Persistent controllers(n = 15)	Transient controllers (n = 15)
*Sex, male*	15 (50%)	26 (87%)	17 (85%)	19 (63%)	6 (40%)	9 (60%)
*Age*	37.6 [33.4-42.5]	35.2 [28.2-42.8]	54.3 [50.6-57.1]	47.5 [39.7-56.1]	45.4 [40.2-51.2]	41.6 [38.1-48.4]
*Years since HIV diagnosis*	-	0.11 [0.04-0.47]	19.3 [11.8-26.3]	13.3 [9.6-19.3]	22.4 [17.4-24.9]	17.5 [12.2-20.3]
*Time on ART*	–	–	11.6 [6.9-17.1]	11.4 [8.7-15.6]	–	–
*Debut HIV viral load*	-	61839 [25999-252988]	52000 [10223-248000]	44800 [15100-857000]	100 [50-200]	78 [50-200]
*Sample HIV viral load*	–	61839 [25999-252988]	<20	<20	20 [20-50]	40 [20-50]
*Debut CD4*	-	363 [217-543]	72 [30.25-177.25]	520 [193-694]	957 [740-1200]	855 [624-1316]
*Nadir CD4*	–	–	18 [12.25-57.25]	184 [59-369]	523 [407-669]	432 [317-624]
*Sample CD4*	860 [764-1023]	363 [217-543]	203 [170-351]	855 [592-1124]	896 [622-1081]	702 [429-890]
*CD4/CD8*	1.68 [1.4-2.09]	N/A	0.3 [0.2-0.5]	1.13 [0.64-1.46]	1.15 [0.6-2]	0.75 [0.5-1.45]
*ART regimen*	-	-	– Integrase inhibitor: 10. – Reverse transcriptase inhibitor: 3. – Others: 7.	– Integrase inhibitor: 23. – Reverse transcriptase inhibitor: 4. – Others: 3.	-	-

Quantitative variables are expressed as the median and Q1-Q3. Qualitative variables are expressed as the number of cases and percentages.

### 3.2 Global metabolic alterations in HIV infection

As a global overview, we first compared all HIV positive participants (n=110) against HIV negative controls. The volcano plot (**Figure 1A**) identified 43 metabolites significantly altered (FDR < 0.05, |FC| > 1.5), of which 33 were up regulated and 10 were down regulated in the HIV positive group. Among the most notable increases were 3 hydroxystearic acid (log2FC = 1.77), glutamic acid (log2FC = 1.23) and beta hydroxylauric acid (log2FC = 1.01). Conversely, the most pronounced decreases included PC(33:2) (log2FC = –0.62), PC(38:6) (log2FC = –0.60) and glycerol (log2FC = –0.76). sPLS DA score plots (**Figure 1B**) showed partial separation between groups along the first two components, which together explained 14.3% of the total variance (component 1: 10.6 %, component 2: 3.7 %).

**Figure 1:**
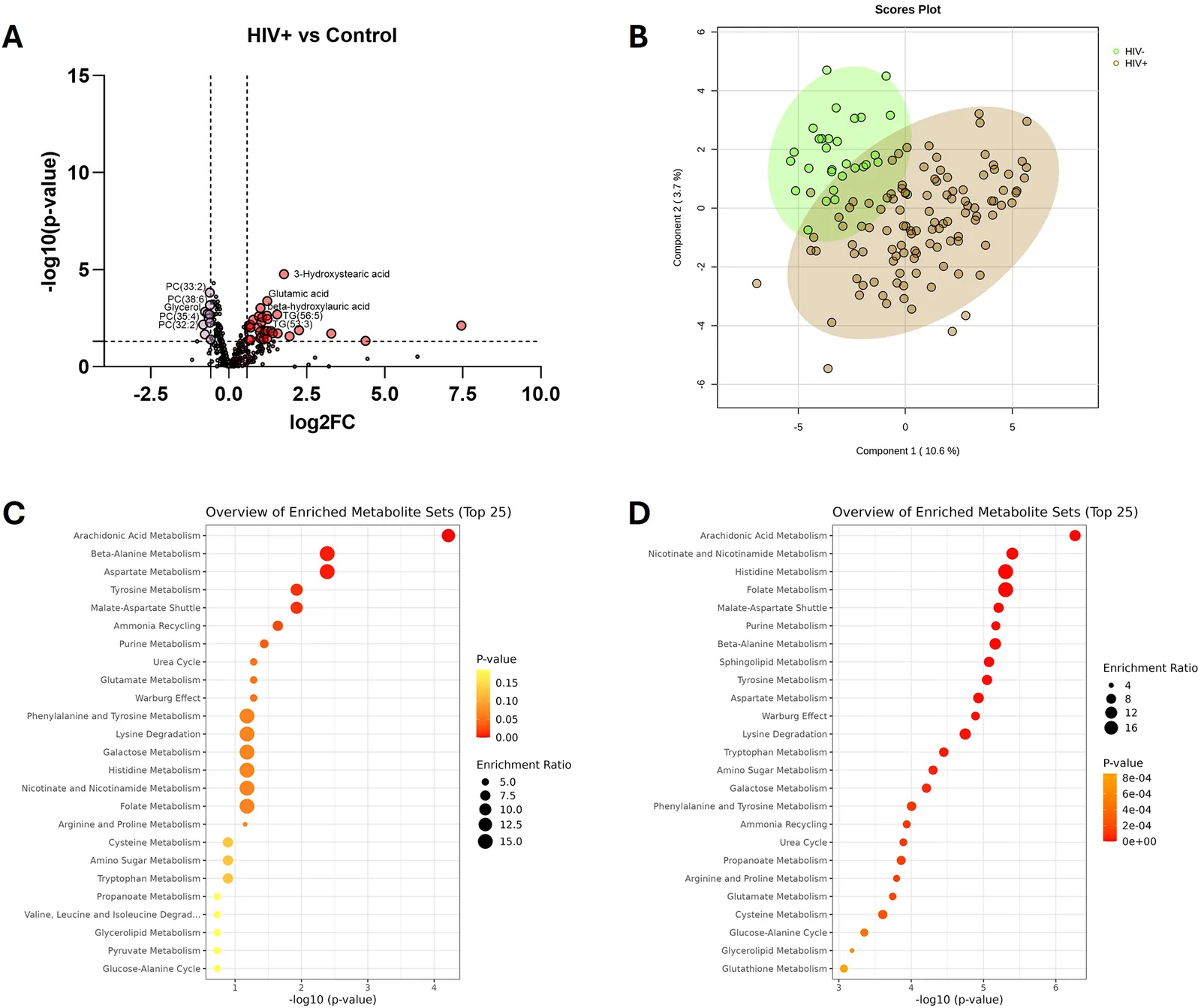
Global plasma metabolomic alterations in HIV infection. **(A)** Volcano plot showing differentially abundant metabolites between HIV-positive participants and healthy controls (FDR < 0.05, |FC| > 1.5). Positive log2 fold change indicates metabolites upregulated; negative log2 fold change indicates downregulated metabolites. **(B)** sPLS-DA score plot showing metabolomic discrimination between groups. **(C)** Over-representation analysis (ORA) of significantly altered metabolites. **(D)** Quantitative enrichment analysis (QEA) of the complete metabolite dataset, highlighting significantly perturbed metabolic pathways.

At the pathway level, ORA (**Figure 1C**) revealed that only arachidonic acid metabolism was significantly enriched (FDR = 0.00596). In contrast, QEA (**Figure 1D**) identified 38 significantly enriched pathways (FDR < 0.05). The most prominent were arachidonic acid metabolism (FDR = 4.18×10^−5^), nicotinate and nicotinamide metabolism (FDR = 7.57×10^−5^) and the malate aspartate shuttle (FDR = 7.57×10^−5^). These global findings were then dissected across the clinical spectrum in the following sections.

### 3.3 Metabolic reprogramming along the ART treated spectrum

Volcano plot analysis of treatment-naive individuals versus controls (**Figure 2A**) revealed 142 significantly altered metabolites, with 66 up-regulated and 76 down-regulated in the naive group. The most pronounced changes included profound decreases in sphingomyelins (SM(d18:1/23:1), log2FC = –0.98; SM(d18:1/21:0), log2FC = –0.86; SM(d18:1/14:1), log2FC = –0.94) and phosphatidylcholines (PC(33:2), log2FC = –0.95), together with marked increases in triglycerides (TG(56:5), log2FC = 2.64; TG(52:3), log2FC = 1.96). sPLS-DA score plots (**Figure 2B**) demonstrated distinct clustering (component 1: 18.4 %, component 2: 7.3 %), with controls positioned in the upper right quadrant and naive individuals in the lower left. ORA (**Figure 2C**) did not identify any pathway with FDR < 0.05. However, QEA (**Figure 2D**) revealed 47 significantly enriched pathways (FDR < 0.05). The most highly enriched was sphingolipid metabolism (FDR = 3.41×10^−14^), followed by transfer of acetyl groups into mitochondria (FDR = 8.02×10^−8^) and arachidonic acid metabolism (FDR = 1.90×10^−7^).

**Figure 2:**
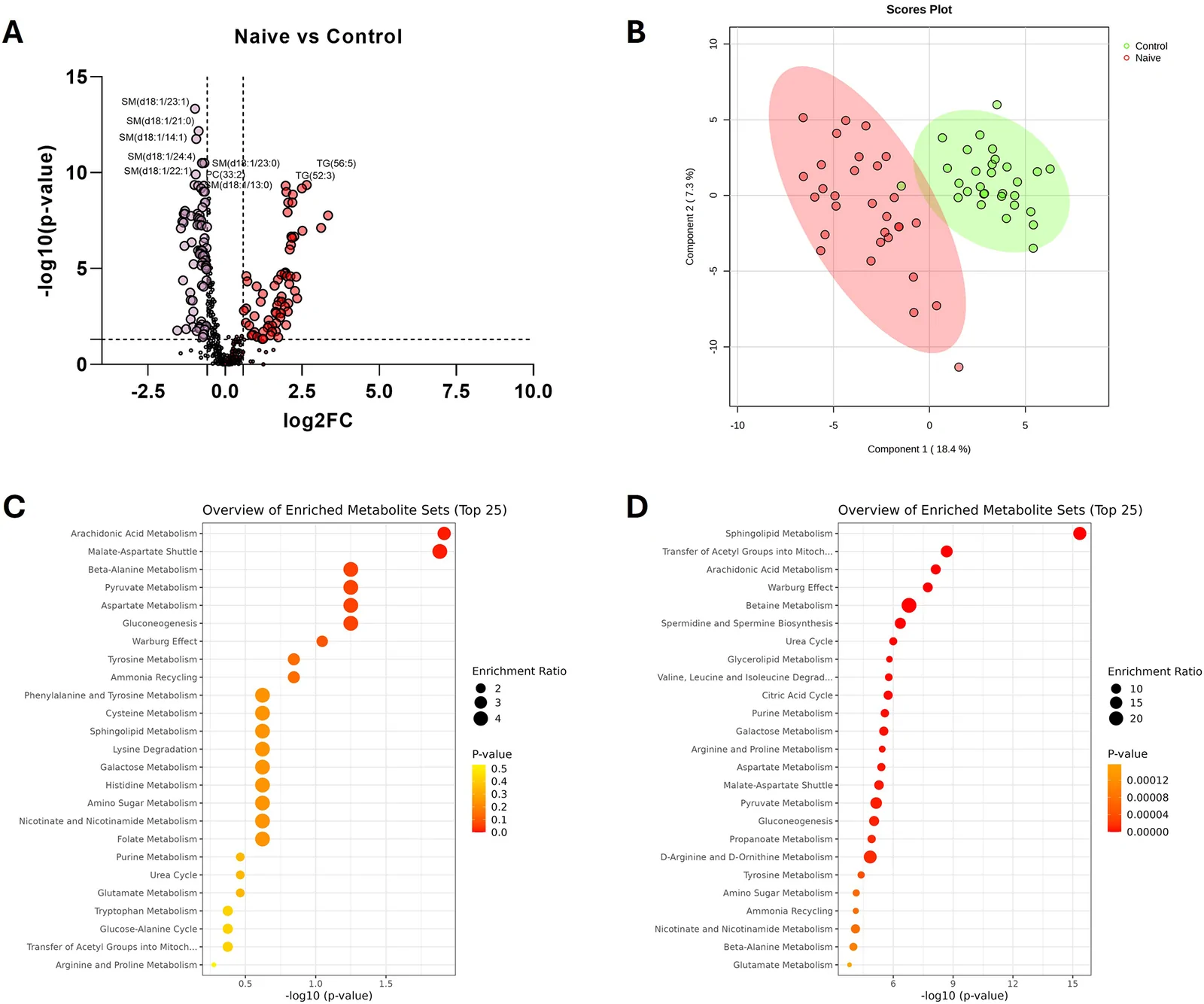
Plasma metabolomic alterations in treatment-naive individuals compared with healthy controls. **(A)** Volcano plot showing differentially abundant metabolites between HIV-positive participants and healthy controls (FDR < 0.05, |FC| > 1.5). Positive log2 fold change indicates metabolites upregulated; negative log2 fold change indicates downregulated metabolites. **(B)** sPLS-DA score plot showing metabolomic discrimination between groups. **(C)** Over-representation analysis (ORA) of significantly altered metabolites. **(D)** Quantitative enrichment analysis (QEA) of the complete metabolite dataset, highlighting significantly perturbed metabolic pathways.

INR participants versus controls (**Figure 3A**) exhibited 114 significantly altered metabolites, with 62 up-regulated and 52 down-regulated in the INR group. The volcano plot showed extreme elevations of eicosanoids: 5-HETE (log2FC = 9.90), 12-HETE (log2FC = 6.61), 9-OxoODE (log2FC = 6.81) and 13-OxoODE (log2FC = 8.47). Conversely, multiple phosphatidylcholines, citric acid (log2FC = –0.81), α-ketoglutarate (log2FC = –1.67) and fructose (log2FC = –2.37) were significantly decreased. sPLS-DA score plots (**Figure 3B**) demonstrated clear group separation (component 1: 16.7 %, component 2: 6.6 %). ORA (**Figure 3C**) did not reach significance for any pathway after FDR correction. In striking contrast, QEA (**Figure 3D**) demonstrated an enrichment of 59 pathways (all FDR < 0.05). The most significant was the urea cycle (FDR = 2.94×10^−9^), followed by amino sugar metabolism (FDR = 2.94×10^−9^) and arginine and proline metabolism (FDR = 2.94×10^−9^).

**Figure 3:**
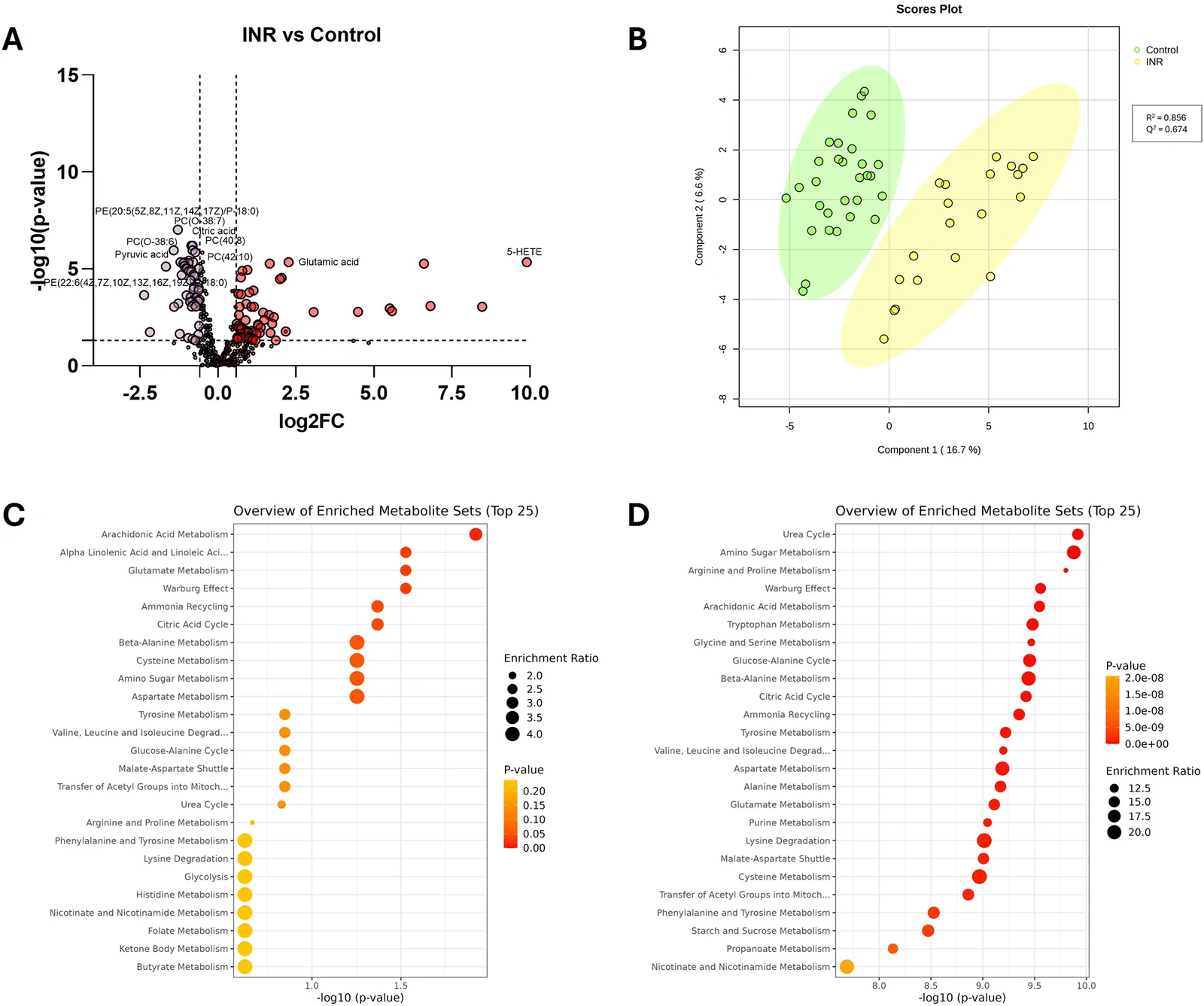
Plasma metabolomic alterations in immune non-responders compared with healthy controls. **(A)** Volcano plot showing differentially abundant metabolites between HIV-positive participants and healthy controls (FDR < 0.05, |FC| > 1.5). Positive log2 fold change indicates metabolites upregulated; negative log2 fold change indicates downregulated metabolites. **(B)** sPLS-DA score plot showing metabolomic discrimination between groups. **(C)** Over-representation analysis (ORA) of significantly altered metabolites. **(D)** Quantitative enrichment analysis (QEA) of the complete metabolite dataset, highlighting significantly perturbed metabolic pathways.

In contrast to INRs, immune responders versus controls displayed a markedly attenuated metabolic signature. The volcano plot (**Figure 4A**) revealed only 4 significantly altered metabolites: increased 5-HETE (log2FC = 1.67) and dihomo-gamma-linolenic acid (log2FC = 0.80), and decreased maltose (log2FC = –0.82) and 9, 10-DHOME (log2FC = –1.31), corresponding to 2 up-regulated and 2 down-regulated metabolites. sPLS-DA score plots (**Figure 4B**) revealed only modest separation (component 1: 5.7 %, component 2: 3.1 %). ORA (**Figure 4C**) yielded no significantly enriched pathways, and QEA (**Figure 4D**) identified only 3 pathways with FDR < 0.05: starch and sucrose metabolism (FDR = 0.000121), arachidonic acid metabolism (FDR = 0.000359) and alpha-linolenic and linoleic acid metabolism (FDR = 0.0326).

**Figure 4:**
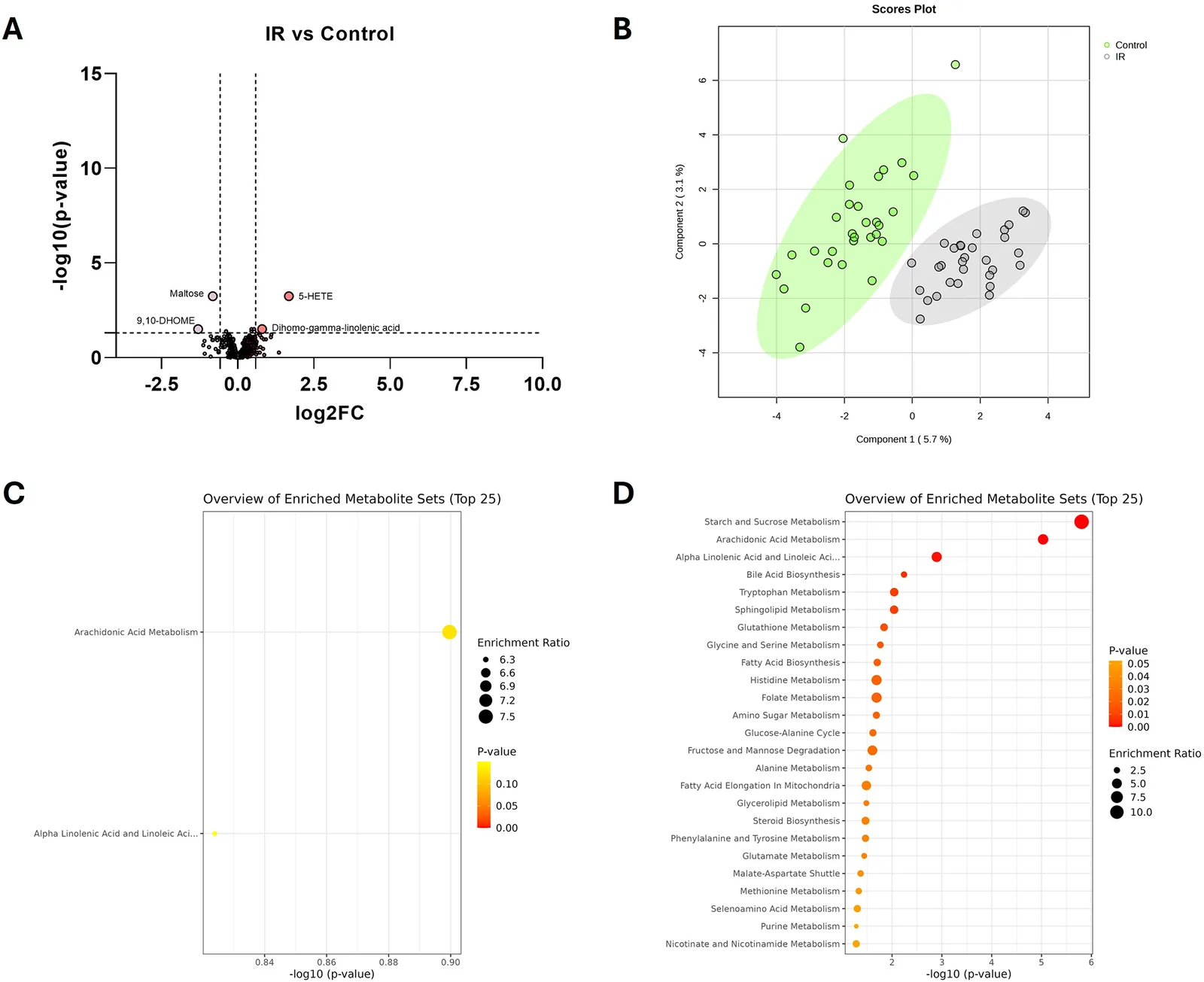
Plasma metabolomic alterations in immune responders compared with healthy controls. **(A)** Volcano plot showing differentially abundant metabolites between HIV-positive participants and healthy controls (FDR < 0.05, |FC| > 1.5). Positive log2 fold change indicates metabolites upregulated; negative log2 fold change indicates downregulated metabolites. **(B)** sPLS-DA score plot showing metabolomic discrimination between groups. **(C)** Over-representation analysis (ORA) of significantly altered metabolites. **(D)** Quantitative enrichment analysis (QEA) of the complete metabolite dataset, highlighting significantly perturbed metabolic pathways.

### 3.4 Metabolic features of natural viral control

PC individuals versus controls showed 28 significantly altered metabolites in the volcano plot (**Figure 5A**), with 23 up-regulated and 5 down-regulated in the PC group. The most notable increases were linoleylcarnitine (log2FC = 2.09), octadecenoylcarnitine (log2FC = 1.85), lactate (log2FC = 1.07), ornithine (log2FC = 0.95), LPC(16:0) (log2FC = 0.88), LPC(18:0) (log2FC = 1.04), palmitoylcarnitine (log2FC = 1.08), aspartate (log2FC = 1.15) and stearoylcarnitine (log2FC = 0.96). Conversely, glycerol (log2FC = –1.15), cis-8, 11, 14, 17-eicosatetraenoic acid (log2FC = –1.15) and eicosapentaenoic acid (log2FC = –1.15) were decreased. sPLS-DA score plots (**Figure 5B**) demonstrated distinct clustering (component 1: 9.3 %, component 2: 5.8 %). ORA (**Figure 5C**) identified 4 significantly enriched pathways, including the urea cycle (FDR = 0.0291), glutamate metabolism (FDR = 0.0291), the Warburg effect (FDR = 0.0291) and arginine and proline metabolism (FDR = 0.0476). QEA (**Figure 5D**) confirmed and extended these findings, revealing 48 significantly enriched pathways (FDR < 0.05). The most highly enriched were pyruvate metabolism (FDR = 8.26×10^−7^), the Warburg effect (FDR = 1.14×10^−6^) and the urea cycle (FDR = 1.14×10^−6^).

**Figure 5:**
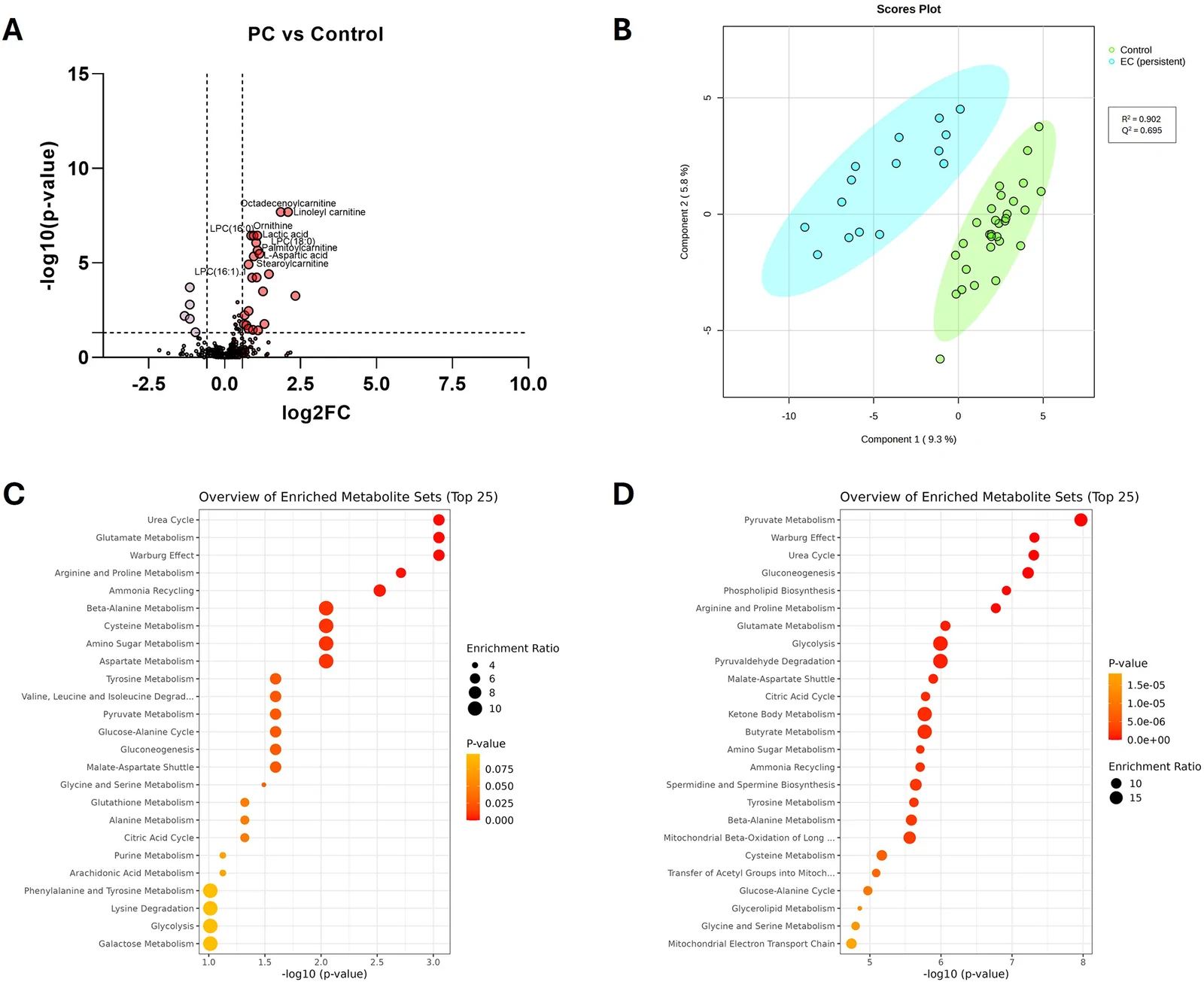
Plasma metabolomic alterations in persistent elite controllers compared with healthy controls. **(A)** Volcano plot showing differentially abundant metabolites between HIV-positive participants and healthy controls (FDR < 0.05, |FC| > 1.5). Positive log2 fold change indicates metabolites upregulated; negative log2 fold change indicates downregulated metabolites. **(B)** sPLS-DA score plot showing metabolomic discrimination between groups. **(C)** Over-representation analysis (ORA) of significantly altered metabolites. **(D)** Quantitative enrichment analysis (QEA) of the complete metabolite dataset, highlighting significantly perturbed metabolic pathways.

TC individuals versus controls exhibited 68 significantly altered metabolites in the volcano plot (**Figure 6A**), with 38 up-regulated and 30 down-regulated in the TC group. The most prominent increases were pyruvate (log2FC = 2.61), lactate (log2FC = 1.53), aspartate (log2FC = 1.36), ornithine (log2FC = 0.99), stearoylcarnitine (log2FC = 0.84) and palmitoylcarnitine (log2FC = 1.20). Decreases were observed in glycerol (log2FC = –1.41), PC(40:8) (log2FC = –0.82) and PC(38:4) (log2FC = –1.09). sPLS-DA score plots (**Figure 6B**) indicated clear group discrimination (component 1: 11.4 %, component 2: 4.7 %). ORA (**Figure 6C**) was not significant for any pathway after FDR correction. However, QEA (**Figure 6D**) revealed 43 significantly enriched pathways (FDR < 0.05). The most significant were the urea cycle (FDR = 6.46×10^−10^), beta-alanine metabolism (FDR = 6.46×10^−10^), tyrosine metabolism (FDR = 6.46×10^−10^), pyruvate metabolism (FDR = 6.46×10^−10^) and ammonia recycling (FDR = 2.37×10^−9^).

**Figure 6:**
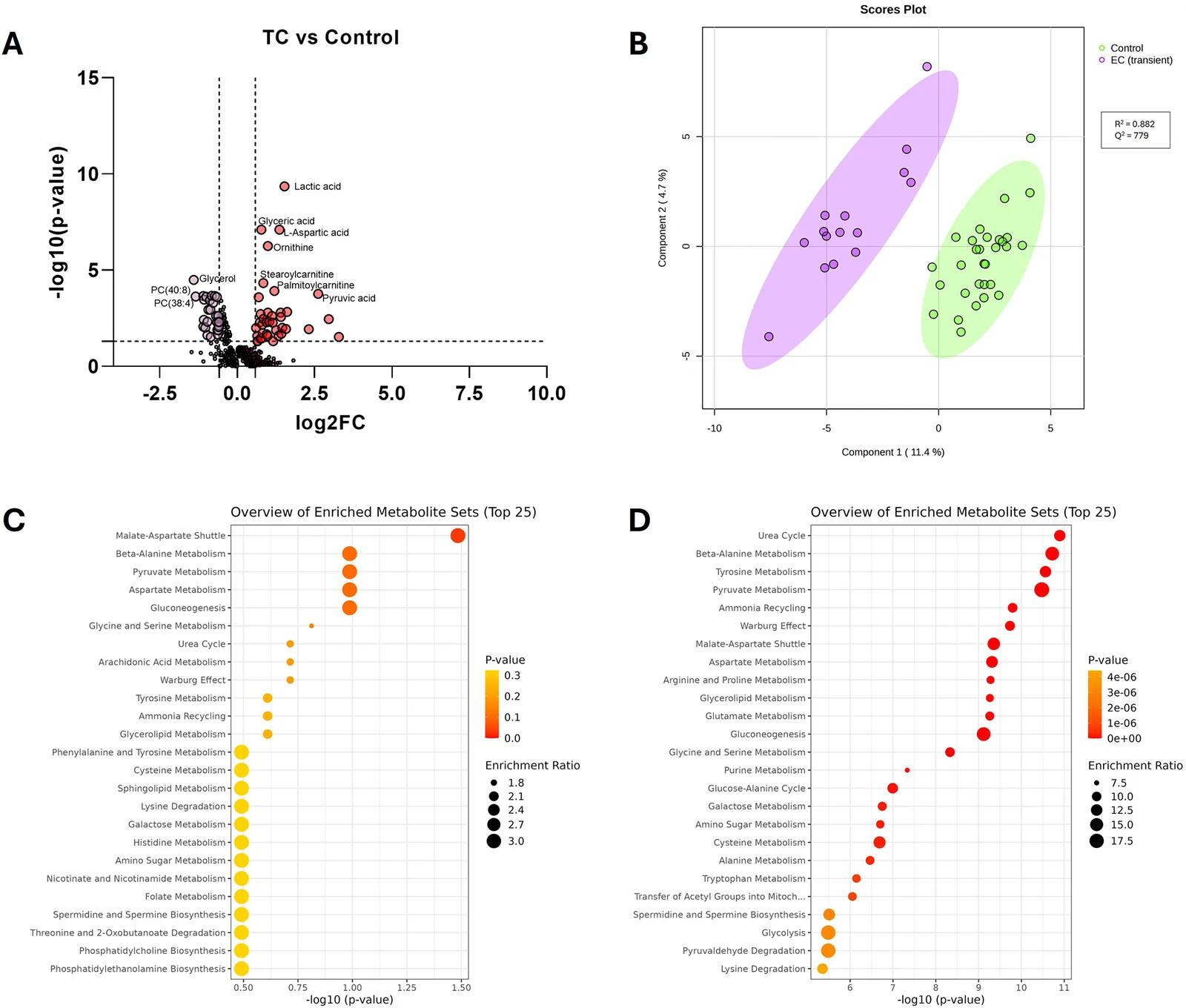
Plasma metabolomic alterations in transient elite controllers compared with healthy controls. **(A)** Volcano plot showing differentially abundant metabolites between HIV-positive participants and healthy controls (FDR < 0.05, |FC| > 1.5). Positive log2 fold change indicates metabolites upregulated; negative log2 fold change indicates downregulated metabolites. **(B)** sPLS-DA score plot showing metabolomic discrimination between groups. **(C)** Over-representation analysis (ORA) of significantly altered metabolites. **(D)** Quantitative enrichment analysis (QEA) of the complete metabolite dataset, highlighting significantly perturbed metabolic pathways.

### 3.5 Complementary pairwise comparisons

To further characterize phenotype specific metabolic signatures beyond the shared alterations common to multiple HIV-positive groups, we performed a series of complementary analyses. For each pairwise comparison between two clinical phenotypes (e.g., INR vs IR, IR vs PC, PC vs TC), we first identified the lists of significantly altered metabolites for each group relative to healthy controls (FDR < 0.05, |FC| > 1.5). A Venn diagram was then constructed to visualize the overlap between the two groups (**Supplementary Figure 1**). Metabolites unique to each phenotype were extracted and subjected to Over Representation Analysis (ORA) to explore pathway enrichment specifically associated with each clinical condition. Quantitative Enrichment Analysis (QEA) was not performed on these filtered metabolite sets, as the algorithm requires the complete dataset of all measured metabolites to reliably assess global pathway perturbation.

Comparison of the metabolite lists from INR (114 metabolites) and IR (4 metabolites) revealed an overlap of 3 shared metabolites (**Supplementary Figure 1A**). The INR group retained 111 unique metabolites, while the IR group had only 1 unique metabolite (9, 10-DHOME), precluding pathway analysis for the IR-specific set. ORA performed on the 111 INR-specific metabolites identified several pathways with nominal raw p-values below 0.05, although none reached significance after FDR correction. The most prominent were glutamate metabolism (Raw p = 0.0245, 4 hits), the Warburg effect (Raw p = 0.0245, 4 hits), ammonia recycling (Raw p = 0.0368, 3 hits) and the citric acid cycle (Raw p = 0.0368, 3 hits). Due to the presence of only a single unique metabolite in IR, ORA could not be performed for this group.

When comparing the metabolite lists of IR (4 metabolites) and PC (28 metabolites), no metabolites were shared between the two phenotypes (**Supplementary Figure 1B**). ORA on the 4 IR-specific metabolites yielded no significant pathway enrichment (raw p > 0.05 for all pathways). In contrast, ORA on the 28 PC-specific metabolites revealed several significantly enriched pathways (FDR < 0.05), including the urea cycle (FDR = 0.0291, 4 hits), glutamate metabolism (FDR = 0.0291, 4 hits), the Warburg effect (FDR = 0.0291, 4 hits) and arginine and proline metabolism (FDR = 0.0476, 4 hits).

The metabolite lists of PC (28 metabolites) and TC (68 metabolites) showed an overlap of 13 shared metabolites, leaving 14 unique to PC and 55 unique to TC (**Supplementary Figure 1C**). ORA of the 14 PC-specific metabolites did not yield any pathway with FDR < 0.05; nominal raw p-values were observed for alpha linolenic and linoleic acid metabolism (Raw p = 0.0289), ketone body metabolism, butyrate metabolism, mitochondrial electron transport chain and phytanic acid peroxisomal oxidation (each with Raw p = 0.0485). ORA of the 55 TC-specific metabolites also yielded no statistically significant pathways after FDR correction.

### 3.6 Global phenotype specific metabolite signatures

Finally, we extended the Venn analysis across all five clinical phenotypes (naive, INR, IR, PC and TC) to identify metabolites uniquely altered in each group relative to controls (**Supplementary Figure 1D**). This global comparison revealed 76 metabolites specific to naive, 47 specific to INR, 0 specific to IR, 8 specific to PC and 22 specific to TC. ORA of the 76 naive-specific metabolites identified pyruvate metabolism (raw p = 0.038, 2 hits) and gluconeogenesis (raw p = 0.038, 2 hits) as nominally enriched, although neither reached FDR significance. For the 47 INR-specific metabolites, alpha-linolenic and linoleic acid metabolism showed a nominal enrichment (raw p = 0.0213, 3 hits). ORA could not be performed for IR due to the absence of unique metabolites. ORA of the 8 PC-specific metabolites revealed nominal enrichment of ketone body metabolism (raw p = 0.0264, 1 hit), butyrate metabolism (raw p = 0.0264, 1 hit), mitochondrial electron transport chain (raw p = 0.0264, 1 hit) and phytanic acid peroxisomal oxidation (raw p = 0.0264, 1 hit). ORA of the 22 TC-specific metabolites identified phospholipid biosynthesis as nominally enriched (raw p = 0.0198, 2 hits).

## 4 Discussion

This study provides a comprehensive characterization of plasma metabolomic alterations across the full viroimmunological spectrum of HIV infection, encompassing untreated individuals, ART-treated immune responders (IR), immune non-responders (INR), and elite controllers (ECs), including both persistent (PC) and transient controllers (TC). While previous metabolomic studies have generally focused on isolated or pairwise comparisons between selected HIV clinical phenotypes, our work integrates all major stages of HIV disease progression and viral control within a single analytical framework. This approach allowed us to identify not only metabolic alterations shared across HIV infection, but also phenotype-specific signatures associated with spontaneous viral control, immune recovery after ART, or persistent immune dysfunction.

### 4.1 Global metabolic perturbations associated with HIV infection

When all HIV-positive participants were analyzed together, the predominant metabolic signature was characterized by activation of arachidonic acid metabolism, disruption of amino acid pathways, and broad remodeling of energy metabolism. The marked increase in eicosanoids, including 5-HETE and other oxidized fatty acid derivatives, together with the strong enrichment of arachidonic acid metabolism in both ORA and QEA, is consistent with previous studies showing persistent activation of inflammatory lipid mediators in people with HIV despite virologic control (7). Elevated eicosanoid production has been linked to chronic immune activation, endothelial dysfunction, and increased cardiovascular risk (8, 9).

Interestingly, although arachidonic acid metabolism represented the only pathway reaching statistical significance by ORA, QEA identified nearly forty significantly perturbed pathways, highlighting the importance of considering coordinated changes involving metabolites that individually may not reach significance thresholds. This difference between enrichment methods likely reflects the diffuse metabolic remodeling characteristic of chronic HIV infection, where numerous metabolites exhibit subtle but coordinated changes rather than isolated large alterations.

Another key finding revealed by the QEA was the coordinated perturbation of pathways governing cellular bioenergetics. Besides the strong enrichment of the malate-aspartate shuttle and the Warburg effect, one of the most prominent findings was the alteration of nicotinate and nicotinamide metabolism, suggesting dysregulation of NAD^+^ homeostasis. Given the central role of NAD^+^ as a cofactor linking glycolysis, the tricarboxylic acid (TCA) cycle and mitochondrial oxidative phosphorylation, disturbances in this pathway may reflect increased metabolic demands associated with chronic immune activation (10).

### 4.2 Metabolic phenotype of treatment-naive participants

The treatment-naive group exhibited the most extensive metabolic disruption of all phenotypes, with 142 significantly altered metabolites and a dominant depletion of sphingomyelins and phosphatidylcholines accompanied by marked accumulation of triglycerides and acylcarnitines. QEA identified sphingolipid metabolism as the most strongly perturbed pathway, followed by pathways related to mitochondrial acetyl-group transfer, pyruvate metabolism, gluconeogenesis, and the Warburg effect. These alterations strongly suggest widespread membrane lipid remodeling and impaired mitochondrial fatty acid oxidation. These findings are biologically coherent with the profound metabolic consequences of uncontrolled HIV replication. HIV infection has been shown to interfere with sphingolipid metabolism at several levels (11).

Sphingolipid-enriched lipid rafts in the plasma membrane act as essential platforms for HIV entry, where the envelope glycoprotein gp120 engages raft-associated gangliosides such as GM1 to initiate membrane fusion (12). During active replication, HIV extensively rewires host sphingolipid metabolism to optimize virion assembly and release (13), and our results are consistent with previous clinical reports of reduced sphingomyelins in untreated HIV, which inversely correlate with circulating IP-10 (7), a pro-inflammatory chemokine we have previously shown to be markedly elevated in treatment-naive individuals (14).

Pathway analyses further indicated profound perturbation of mitochondrial metabolism. Transfer of acetyl groups into mitochondria, the malate-aspartate shuttle, pyruvate metabolism and gluconeogenesis were among the most significantly enriched pathways, all of which depend on coordinated mitochondrial activity.

Interestingly, the global Venn analysis identified 76 metabolites uniquely altered in naive individuals, representing the largest phenotype-specific metabolomic signature observed in the study. Although pathway enrichment of these unique metabolites did not remain significant after multiple-testing correction, the nominal enrichment of pyruvate metabolism and gluconeogenesis further reinforces the central role of energy metabolism during uncontrolled HIV replication. These findings suggest that a considerable proportion of the metabolic disturbances observed in untreated infection are associated with active viral replication and become partially normalized following effective antiretroviral therapy, an interpretation that is strongly supported by the markedly attenuated metabolomic profile observed in immune responders discussed below.

### 4.3 Persistent metabolic dysfunction in immune non-responders

Persistent immune activation is considered one of the defining characteristics of INR (15) and constitutes one of the strongest predictors of mortality in people living with HIV despite effective ART (16). Several mechanisms have been proposed to explain this phenomenon, including thymic dysfunction, abnormal immune activation, T-cell exhaustion, microbial translocation, and gut microbiota imbalances (17). Our metabolomic findings suggest that these immunological abnormalities are accompanied by profound alterations in intermediary metabolism that are likely associated with the maintenance of chronic inflammation.

Compared with controls, INRs exhibited an accumulation of 5-HETE, a lipid mediator derived from arachidonic acid metabolism. This points towards enhanced activation of the arachidonic acid cascade, reinforcing the concept of persistent inflammatory signaling. Conversely, the reduction of citric acid, together with the enrichment of pathways involving the TCA cycle and mitochondrial acetyl-group transfer, is compatible with altered mitochondrial metabolism and increased utilization of TCA intermediates. Elevated glutamic acid levels, alongside the strong enrichment of glutamate- and aspartate-related pathways, further suggest intensified amino acid catabolism feeding central carbon metabolism. Finally, regarding the decline in multiple phosphatidylcholine ethers and phosphatidylethanolamines, it has been reported that low levels of these compounds are associated with chronic inflammatory processes (18), once again highlighting this inflammation underlying HIV infection.

Conversely, the reduction of citric acid, together with the enrichment of pathways involving the tricarboxylic acid (TCA) cycle and mitochondrial acetyl-group transfer, is compatible with altered mitochondrial metabolism and increased utilization of TCA intermediates. This metabolic remodeling was accompanied by extensive perturbation of amino acid metabolism, including glutamate, aspartate, alanine, glycine and serine metabolism, branched-chain amino acid degradation, arginine and proline metabolism, the urea cycle and tryptophan metabolism. Collectively, these coordinated metabolic changes suggest increased biosynthetic activity associated with persistent immune activation, a hallmark of immune non-response that has been linked to impaired CD4^+^ T-cell recovery despite suppressive ART (19). Consistent with this, the pronounced CD4/CD8 ratio inversion observed in INR (median 0.3, versus 1.13 in IR) is a recognized marker of persistent immune activation and immunosenescence, and is mechanistically linked to the metabolic alterations described: impaired urea cycle function may limit arginine availability for CD4^+^ T-cell proliferation (20).

The pairwise INR-versus-IR analysis reinforces this interpretation. After removing metabolites shared with IR, 111 metabolites remained unique to INR and were enriched for glutamate metabolism, the Warburg effect, ammonia recycling, and the citric acid cycle. Thus, the metabolic distinction between successful and unsuccessful immune reconstitution appears to be driven less by residual inflammatory lipids and more by persistent defects in mitochondrial and amino acid metabolism.

### 4.4 Near-normalization of metabolism in immune responders

In sharp contrast to the extensive metabolic disturbances observed in untreated individuals and immune non-responders, immune responders exhibited a remarkably preserved plasma metabolome. Only four metabolites differed significantly from healthy controls, and pathway analyses identified just three significantly enriched pathways. These findings suggest that successful immune reconstitution following long-term ART is accompanied by an almost complete normalization of systemic metabolism, reinforcing the concept that metabolic homeostasis is closely linked to the combination of viral suppression and adequate CD4^+^ T-cell recovery (21).

Previous metabolomic studies have reported that ART only partially reverses the metabolic alterations induced by HIV infection (22). However, by specifically separating immune responders from immune non-responders, our study demonstrates that much of the residual metabolic dysfunction may actually arise from individuals who fail to reconstitute their immune system adequately. This distinction is clinically relevant because it suggests that persistent metabolic abnormalities are not an inevitable consequence of lifelong ART but are instead associated with ongoing immunopathology in a subset of treated individuals.

Among the few metabolites that remained, the persistent elevation of 5-HETE indicates that complete resolution of inflammatory lipid signaling may not occur even after prolonged suppressive ART and successful CD4^+^ T-cell recovery (23). Chronic low-grade inflammation has become increasingly recognized as a characteristic feature of treated HIV infection and has been implicated in the elevated incidence of cardiovascular disease, neurocognitive impairment and other non-AIDS comorbidities (24). Therefore, our results support the idea that there is persistent, subtle activation of arachidonic acid metabolism even in clinically well-controlled individuals, a finding reinforced by the fact that arachidonic acid metabolism was one of only three pathways that remained significantly altered.

Interestingly, the other two enriched pathways—starch and sucrose metabolism and α-linolenic/linoleic acid metabolism—suggest that the residual metabolic disturbances in immune responders extend beyond inflammatory lipid mediators. The enrichment of starch and sucrose metabolism, despite the absence of broader alterations in glycolysis, pyruvate metabolism or the Warburg effect, may reflect subtle adaptations in carbohydrate metabolism. Likewise, the enrichment of α-linolenic and linoleic acid metabolism, which has been described in HIV infection despite viral suppression achieved through ART (23), suggests an ongoing remodeling of polyunsaturated fatty acid pathways, which are intimately linked to both membrane lipid homeostasis and the synthesis of downstream lipid mediators, including arachidonic acid-derived eicosanoids.

### 4.5 Metabolic adaptations associated with persistent spontaneous viral control

Elite controllers represent one of the most fascinating clinical phenotypes in HIV research. Unlike ART-treated individuals, these rare patients maintain long-term suppression of viral replication in the absence of antiretroviral therapy, providing a unique natural model of functional cure (25). Although host genetic factors, particularly protective HLA class I alleles, contribute substantially to this phenotype, viral factors also appear to play an important role. In elite controllers, replication-competent HIV-1 proviruses are more frequently integrated into transcriptionally inactive, non-coding regions of the host genome, promoting a state of deep viral latency and reducing the likelihood of spontaneous viral reactivation (26).

Rather than constituting a homogeneous population, elite controllers encompass individuals with markedly different clinical trajectories. Persistent controllers maintain durable virological control for many years, whereas transient controllers eventually lose spontaneous suppression and progress toward chronic infection (27). Understanding the metabolic differences between these two phenotypes may therefore provide valuable insights into the biological mechanisms that determine long-term maintenance or eventual failure of natural viral control.

Elite controllers represent a unique model of functional HIV control, as they maintain undetectable viral loads in the absence of ART. However, accumulating evidence indicates that spontaneous viral control does not necessarily equate to complete immunological homeostasis. Several studies have demonstrated that elite controllers exhibit persistent immune activation, increased inflammatory biomarkers and an elevated risk of non-AIDS comorbidities (28).

Our metabolomic data support this concept. Although the metabolic alterations observed in persistent controllers were substantially less extensive than those of treatment-naive participants, they were clearly distinguishable from healthy controls. The predominant features included increased acylcarnitines, lactate, aspartate and ornithine together with enrichment of pyruvate metabolism, the Warburg effect and the urea cycle, consistent with previous metabolomic evidence indicating that maintenance of spontaneous HIV control is associated with a distinctive immunometabolic profile characterized by regulation of mitochondrial and glycolytic pathways (29). Acylcarnitines facilitate mitochondrial transport of long-chain fatty acids and their accumulation has been associated both with incomplete β-oxidation and with increased mitochondrial metabolic flux (30), which likely reflects increased metabolic flexibility required to sustain effective antiviral immune responses. Growing evidence indicates that highly functional antiviral T-cells exhibit considerable metabolic plasticity, enabling rapid switching between glycolysis and oxidative phosphorylation according to energetic requirements (31), rather than relying exclusively on one metabolic program.

### 4.6 Pre-loss metabolic state of transient controllers

One of the most distinctive aspects of the present study is the inclusion of plasma samples obtained from transient controllers before the loss of virological control. This unique cohort provides a rare opportunity to investigate metabolic alterations that precede HIV progression rather than merely reflecting its consequences.

Compared with persistent controllers, transient controllers exhibited substantially greater metabolic perturbation, with more than twice the number of significantly altered metabolites relative to healthy controls. QEA identified significant enrichment of pyruvate metabolism, β-alanine metabolism, tyrosine metabolism, ammonia recycling and the urea cycle. These pathways collectively indicate extensive remodeling of central carbon and nitrogen metabolism, supporting increased biosynthetic demand within activated immune cells.

Unlike persistent controllers, transient controllers also exhibited a larger reduction in phosphatidylcholines together with decreased glycerol concentrations. Progressive phospholipid depletion may have important biological consequences beyond membrane remodeling. Phosphatidylcholines serve as major reservoirs of polyunsaturated fatty acids that can subsequently be converted into pro-inflammatory lipid mediators (32). Therefore, enhanced phospholipid turnover may both reflect and contribute to increasing inflammatory activity during the period immediately preceding loss of viral control.

Interestingly, the complementary Venn analyses identified fifty-five metabolites unique to transient controllers when compared with persistent controllers. Although pathway enrichment did not reach statistical significance after correction for multiple comparisons, the absence of statistically significant ORA results for transient controller-specific metabolites should not necessarily be interpreted as evidence of biological irrelevance. The relatively small number of phenotype-specific metabolites substantially limits statistical power after multiple-testing correction.

Collectively, our data support a model in which transient controllers occupy an intermediate immunometabolic state between durable spontaneous control and progressive HIV infection. Importantly, the median interval between sampling and virological rebound was 30 [14-48] months, indicating that these metabolic alterations are not immediate predictors of impending loss of control. Rather, they appear to represent a stable metabolic trait that precedes virological failure by several years. These findings raise the possibility that metabolomic profiling could eventually contribute to identifying elite controllers at increased risk of losing spontaneous viral suppression before conventional clinical markers become abnormal.

### 4.7 Biological synthesis and implications

Across the entire viroimmunological spectrum, three interconnected metabolic themes emerged. First, arachidonic acid metabolism remained activated in virtually all HIV-positive phenotypes, indicating that inflammatory lipid signaling persists independently of viral suppression strategy. Second, pathways related to glycolysis, pyruvate metabolism, and the Warburg effect progressively intensified from controls to naive participants, INRs, and TCs, supporting the concept that chronic HIV-associated immune activation is tightly linked to aerobic glycolysis. Third, the recurrent enrichment of the urea cycle, glutamate metabolism, and arginine/proline metabolism points to sustained alterations in nitrogen and amino acid handling that may influence T-cell function and immune recovery.

Although each clinical group exhibited a distinct metabolic profile, the complementary pairwise comparisons suggest that HIV infection is better understood as a continuum of metabolic states rather than as a collection of independent phenotypes. Instead of abrupt transitions between groups, the metabolomic data indicate a progressive remodeling associated with viral replication, immune recovery, long-term ART, and spontaneous viral control.

From a biological perspective, this continuum highlights that metabolomic remodeling is associated not exclusively by viral replication but also by the balance between chronic inflammation and immune competence. Consequently, specific metabolic signatures identified along this trajectory may provide mechanistic insight into disease progression while also representing potential biomarkers for monitoring immune recovery and the stability of spontaneous viral control.

Several considerations should be taken into account when interpreting these findings. Sample sizes were modest for some phenotypes, particularly elite controllers, and pathway analyses based on small metabolite subsets should therefore be considered exploratory. Although groups differed in age and sex distribution, primary analyses were adjusted for both variables. Participants were recruited from different centers according to phenotype; however, samples were processed using standardized protocols and labelled internal standards were used to monitor analytical performance. Detailed longitudinal ART histories were unavailable, precluding adjustment for cumulative drug-class exposure. Nevertheless, IR and INR participants had nearly identical ART duration and overlapping treatment eras, making major systematic differences in overall treatment exposure less likely.

The cross-sectional design limits inference on within-individual metabolic trajectories, although the availability of samples from transient controllers before virological rebound represents a distinctive strength and provides a rationale for future longitudinal validation. In addition, the targeted and semi-targeted platforms capture a defined fraction of the circulating metabolome, and plasma profiles reflect systemic rather than tissue-specific metabolism. Finally, unmeasured factors such as diet, physical activity, gut microbiome composition, and chronic co-infections, including CMV, may contribute to metabolic variability. Accordingly, the identified signatures should be validated in larger independent and longitudinal cohorts.

This study provides an integrated characterization of the plasma metabolome across the full viroimmunological spectrum of HIV infection within a single analytical framework. Distinct metabolic signatures differentiated each clinical phenotype: treatment-naive individuals showed the most extensive disruption, dominated by sphingolipid depletion and triglyceride accumulation; immune non-responders exhibited a profile combining pronounced eicosanoid elevation with sustained alterations of nitrogen and mitochondrial metabolism; immune responders displayed near-complete metabolic normalization; and elite controllers presented a distinctive immunometabolic profile, with transient controllers showing substantially broader perturbation of central carbon and nitrogen metabolism than persistent controllers before the loss of spontaneous viral control.

Three findings have particular translational relevance. The persistence of arachidonic acid metabolism activation across virtually all phenotypes, including successfully treated individuals, indicates that inflammatory lipid signaling is not fully resolved by viral suppression alone. The near-normal metabolome of immune responders, contrasted with the extensive alterations in immune non-responders on comparable ART duration, suggests that residual metabolic dysfunction in treated HIV infection is associated principally with inadequate immune reconstitution rather than by antiretroviral exposure per se. And the metabolic perturbations detected in transient controllers before virological rebound raise the possibility that metabolomic profiling could contribute to identifying elite controllers at risk of losing spontaneous control.

These findings provide a framework for understanding the metabolic heterogeneity associated with distinct viroimmunological outcomes of HIV infection. Validation in larger, demographically balanced and longitudinal cohorts will be important to determine the reproducibility and clinical relevance of these signatures, particularly their potential utility for monitoring immune recovery, identifying loss of immunological stability, and informing targeted therapeutic strategies beyond viral suppression.

## Data Availability

The raw data has been made publicly available in BioStudies (https://www.ebi.ac.uk/biostudies/), under accession number S-BSST3409).

## Supplementary material

Supplementary Figure 1 Venn diagrams showing the overlap of significantly altered metabolites (FDR < 0.05, |FC| > 1.5) between clinical phenotypes. **(A)** INR versus IR. **(B)** IR versus PC. **(C)** PC versus TC. **(D)** All HIV‑positive groups (naive, INR, IR, PC and TC).

## References

[1] Deeks SG Lewin SR Havlir DV . The end of AIDS: HIV infection as a chronic disease. Lancet. (2013) 382:1525–33. doi: 10.1016/S0140-6736(13)61809-7 24152939 PMC4058441

[2] Fan L Li P Yu A Liu D Wang Z Wu Y . Prevalence of and prognosis for poor immunological recovery by virally suppressed and aged HIV-infected patients. Front Med (Lausanne). (2023) 10. doi: 10.3389/fmed.2023.1259871 37928477 PMC10625403

[3] Okulicz JF Lambotte O . Epidemiology and clinical characteristics of elite controllers. Curr Opin HIV AIDS. (2011) 6:163–8. doi: 10.1097/COH.0b013e328344f35e 21502920

[4] Ding Y Lin H Chen X Zhu B Xu X Xu X . Comprehensive metabolomics profiling reveals common metabolic alterations underlying the four major non-communicable diseases in treated HIV infection. EBioMedicine. (2021) 71:103548. doi: 10.1016/j.ebiom.2021.103548 34419928 PMC8385138

[5] Cassol E Misra V Holman A Kamat A Morgello S Gabuzda D . Plasma metabolomics identifies lipid abnormalities linked to markers of inflammation, microbial translocation, and hepatic function in HIV patients receiving protease inhibitors. BMC Infect Dis. (2013) 13:203. doi: 10.1186/1471-2334-13-203 23641933 PMC3655873

[6] Sobrino-Vegas P Gutiérrez F Berenguer J Labarga P García F Alejos-Ferreras B . La cohorte de la red española de investigación en sida y su biobanco: organización, principales resultados y pérdidas al seguimiento. Enferm Infecc Microbiol Clin. (2011) 29:645–53. doi: 10.1016/j.eimc.2011.06.002 21820763

[7] Peltenburg NC Schoeman JC Hou J Mora F Harms AC Lowe SH . Persistent metabolic changes in HIV-infected patients during the first year of combination antiretroviral therapy. Sci Rep. (2018) 8:16947. doi: 10.1038/s41598-018-35271-0 30446683 PMC6240055

[8] Dennis EA Norris PC . Eicosanoid storm in infection and inflammation. Nat Rev Immunol. (2015) 15:511–23. doi: 10.1038/nri3859 26139350 PMC4606863

[9] Piper K Garelnabi M . Eicosanoids: Atherosclerosis and cardiometabolic health. J Clin Transl Endocrinol. (2020) 19:100216. doi: 10.1016/j.jcte.2020.100216 32071878 PMC7013337

[10] Palmer CS Ostrowski M Balderson B Christian N Crowe SM . Glucose metabolism regulates T cell activation, differentiation, and functions. Front Immunol. (2015) 6. doi: 10.3389/fimmu.2015.00001 25657648 PMC4302982

[11] Schneider-Schaulies J Schneider-Schaulies S . Sphingolipids in viral infection. Biol Chem. (2015) 396:585–95. doi: 10.1515/hsz-2014-0273 25525752

[12] Thomas S Samuel SV Hoch A Syphurs C Diray-Arce J . The implication of sphingolipids in viral infections. Int J Mol Sci. (2023) 24:17303. doi: 10.3390/ijms242417303 38139132 PMC10743733

[13] Tomishige N Bin Nasim M Murate M Pollet B Didier P Godet J . HIV-1 Gag targeting to the plasma membrane reorganizes sphingomyelin-rich and cholesterol-rich lipid domains. Nat Commun. (2023) 14:7353. doi: 10.1038/s41467-023-42994-w 37990014 PMC10663554

[14] Álvarez H Gutiérrez-Valencia A Mariño A Saborido-Alconchel A Calderón-Cruz B Pérez-González A . IP-10 and MIG are sensitive markers of early virological response to HIV-1 integrase inhibitors. Front Immunol. (2023) 14. doi: 10.3389/fimmu.2023.1257725 37920466 PMC10619723

[15] Silvestre C Gross A Makinson A . Immune non‐response despite effective antiretroviral therapy in people living with <scp>HIV</scp>: A review of potential mechanisms, biomarkers and therapeutic approaches. HIV Med. (2026) 27:498–512. doi: 10.1111/hiv.70197 41639021 PMC13051040

[16] Wada NI Bream JH Martínez-Maza O Macatangay B Galvin SR Margolick JB . Inflammatory biomarkers and mortality risk among HIV-suppressed men: A multisite prospective cohort study. Clin Infect Dis. (2016) 63:984–90. doi: 10.1093/cid/ciw409 27343547 PMC5019283

[17] Pang X He Q Huang J Tang K Fang N Xie H . Characteristics and influencing factors of immunological non-responders in HIV-1-infected patients receiving antiretroviral therapy: a cross-sectional study in Guangxi. Sci Rep. (2024) 14:27739. doi: 10.1038/s41598-024-79449-1 39532976 PMC11558013

[18] Bozelli JC Azher S Epand RM . Plasmalogens and chronic inflammatory diseases. Front Physiol. (2021) 12. doi: 10.3389/fphys.2021.730829 34744771 PMC8566352

[19] Espineira S Flores-Piñas M Chafino S Viladés C Negredo E Fernández-Arroyo S . Multi-omics in HIV: searching insights to understand immunological non-response in PLHIV. Front Immunol. (2023) 14. doi: 10.3389/fimmu.2023.1228795 37649488 PMC10465175

[20] Zea AH Rodriguez PC Culotta KS Hernandez CP DeSalvo J Ochoa JB . l-Arginine modulates CD3ζ expression and T cell function in activated human T lymphocytes. Cell Immunol. (2004) 232:21–31. doi: 10.1016/j.cellimm.2005.01.004 15922712

[21] Virseda-Berdices A Martín-Escolano R Berenguer J González-García J Brochado-Kith O Rojo D . Plasma metabolomic profile is near-normal in people with HIV on long-term suppressive antiretroviral therapy. Front Cell Infect Microbiol. (2024) 14. doi: 10.3389/fcimb.2024.1340610 38550617 PMC10972849

[22] Deme P Rubin LH Yu D Xu Y Nakigozi G Nakasujja N . Immunometabolic reprogramming in response to HIV infection is not fully normalized by suppressive antiretroviral therapy. Viruses. (2022) 14:1313. doi: 10.3390/v14061313 35746785 PMC9228482

[23] Deme P Moniruzzaman M Moore D Heaton R Ellis R Letendre S . Association of plasma eicosanoid levels with immune, viral, and cognitive outcomes in people with HIV. Neurology. (2022) 99(12):e1251–e1264. doi: 10.1212/WNL.0000000000200945 35851253 PMC9576290

[24] Babu H Ambikan AT Gabriel EE Svensson Akusjärvi S Palaniappan AN Sundaraj V . Systemic inflammation and the increased risk of inflamm-aging and age-associated diseases in people living with HIV on long term suppressive antiretroviral therapy. Front Immunol. (2019) 10. doi: 10.3389/fimmu.2019.01965 31507593 PMC6718454

[25] Walker BD Yu XG . Unravelling the mechanisms of durable control of HIV-1. Nat Rev Immunol. (2013) 13:487–98. doi: 10.1038/nri3478 23797064

[26] Hartana CA Yu XG . Immunological effector mechanisms in HIV-1 elite controllers. Curr Opin HIV AIDS. (2021) 16:243–8. doi: 10.1097/COH.0000000000000693 34270465 PMC8373669

[27] Pernas M Tarancón-Diez L Rodríguez-Gallego E Gómez J Prado JG Casado C . Factors leading to the loss of natural elite control of HIV-1 infection. J Virol. (2018) 92(5):e01805-17. doi: 10.1128/JVI.01805-17 29212942 PMC5809746

[28] Crowell TA Hatano H . Clinical outcomes and antiretroviral therapy in ‘elite’ controllers: a review of the literature. J Virus Erad. (2015) 1:72–7. doi: 10.1016/S2055-6640(20)30488-X 27123315 PMC4844069

[29] Tarancon-Diez L Rodríguez-Gallego E Rull A Peraire J Viladés C Portilla I . Immunometabolism is a key factor for the persistent spontaneous elite control of HIV-1 infection. EBioMedicine. (2019) 42:86–96. doi: 10.1016/j.ebiom.2019.03.004 30879922 PMC6491381

[30] McCoin CS Knotts TA Adams SH . Acylcarnitines—old actors auditioning for new roles in metabolic physiology. Nat Rev Endocrinol. (2015) 11:617–25. doi: 10.1038/nrendo.2015.129 26303601 PMC4966159

[31] Chang C-H Curtis JD Maggi LB Faubert B Villarino AV O’Sullivan D . Posttranscriptional control of T cell effector function by aerobic glycolysis. Cell. (2013) 153:1239–51. doi: 10.1016/j.cell.2013.05.016 23746840 PMC3804311

[32] Harwood JL . Polyunsaturated fatty acids: Conversion to lipid mediators, roles in inflammatory diseases and dietary sources. Int J Mol Sci. (2023) 24:8838. doi: 10.3390/ijms24108838 37240183 PMC10218335

